# Comparison of Rhizosphere Fungal Community Changes in Healthy and Yellowing-Leaf-Disease-Affected Areca Palms by High-Throughput Sequencing Technology

**DOI:** 10.3390/jof11110803

**Published:** 2025-11-12

**Authors:** Wenqing Yang, Rui Ma, Ying Wei, Miaomiao Liu, Daojun Zheng, Kai Rui, Shunyi Yang

**Affiliations:** 1 Sanya Institute of Hainan Academy of Agricultural Sciences (Hainan Provincial Research Center of Laboratory Animals), Sanya 572024, China; 13150065246@163.com (W.Y.); daojunzh@163.com (D.Z.); 2 Key Laboratory of Plant Disease and Pest Control of Hainan Province, Haikou 570100, China; 13034999249@163.com (R.M.); nkywy0909@163.com (Y.W.); 17693229659@163.com (M.L.); 3 Institute of Plant Protection, Hainan Academy of Agricultural Sciences (Research Center of Quality Safety and Standards for Agro-Products, Hainan Academy of Agricultural Sciences), Haikou 571100, China; 4 Scientific Observation and Experiment Station of Crop Pests in Haikou, Ministry of Agriculture and Rural Affairs, Haikou 570100, China; 5 College of Plant Protection, Gansu Agricultural University, Lanzhou 730070, China

**Keywords:** yellow leaf disease, areca palm velarivirus 1, areca rhizosphere fungi, community composition, biodiversity

## Abstract

Yellow leaf disease (YLD) has been the most severe disease threatening areca palm, commonly known in areca palm cultivation. However, it has not yet been systematically studied in terms of the relationship between infected plants and the structure of rhizosphere microbial communities. In order to systematically study the impact of YLD on the rhizosphere fungi of the areca palm, we implemented high-throughput sequencing technology to analyze the microbial community structure and diversity under different disease conditions. The results indicate that as the severity of the disease increases, the diversity of the fungal community diminishes, with species abundance and richness initially decreasing before subsequently increasing, while phylogenetic diversity increases, and significant changes occur in the structure of the soil fungal community. At the phylum level, the dominant fungal phyla in the rhizosphere of areca palm are *Ascomycota* and *Basidiomycota*. At the genus level, the dominant genera are *Sarocladium*, *Roussoella*, *Penicillium*, etc., and their relative abundance increases with the severity of the disease. LEfSe analysis revealed that *Archaeorhizomyces*, *Codinaea*, and *Albifimbria* serve as indicator species for healthy areca palms, with their relative abundance trends consistent with changes in Alpha diversity. FUNGuild prediction results indicated that the fungal nutrient type structures of the three rhizosphere samples were highly similar, with saprotrophs being the absolutely dominant type. With the increase in the severity of the disease, the number of harmful fungi in the soil (such as *Plectosphaerella*, *Fusarium*, etc.) increases, thereby limiting the sustainable development of the soil. Network analysis indicates that beneficial microbial communities such as *Stachybotrys* and *Roussoella* exhibit extensive negative interactions. Therefore, the YLD of areca palm significantly alters the structure and diversity of the rhizosphere fungal community. Simultaneously, some beneficial microorganisms may be recruited by the areca rhizosphere to resist the invasion of YLD by improving the rhizosphere environment and enhancing plant immunity, such as *Trechispora*, *Saitozyma*, and *Marasmiellus*. This experiment is expected to provide a theoretical basis for the study of the rhizosphere microecology of the areca palm, the exploration of excellent biocontrol resources, and the green control of YLD in the areca palm.

## 1. Introduction

Areca palm (*Areca catechu* L.) is a perennial evergreen tree belonging to the genus Areca in the family Arecaceae (Palmae), primarily distributed in the tropical and subtropical fringe regions of Asia, Africa, Europe, and Central America [1]. It has become one of the most important economic trees and is widely cultivated due to its various medicinal and edible functions, including anti-fatigue, antioxidant, anti-depressant, anti-inflammatory, analgesic, and diuretic properties [2,3]. Areca palm is native to Malaysia and is primarily distributed in Yunnan, Guangxi, Guangdong, Fujian, Hainan, and Taiwan Province in China. Hainan Province, as the core area for betel nut cultivation in China, accounts for over 95% of both the planting area and total output in the mainland [4]. By the end of 2020, the fruit of the areca palm cultivation in the province had exceeded 150,000 hectares, with a total output value of over 20 billion yuan, making it the primary source of income for more than 2 million farmers in Hainan [5]. It plays a pivotal role in the development of Hainan’s tropical high-efficiency agriculture and the construction of the National Ecological Civilization Pilot Zone [6]. However, the occurrence of pests and diseases has severely impacted the medicinal value and economic benefits of betel nut.

Yellow leaf disease (YLD), caused by Areca palm velarivirus 1 (APV1), is one of the most severe diseases, occurring in all major areca palm production regions worldwide [7]. YLD was first reported in India by Kerala et al. (1914) [8] and was also discovered in Tunchang County, Hainan Province, China, in 1985. As of 2022, the affected area in Hainan Province reached 32 thousand hectares, accounting for 27.39% of the province’s total areca palm cultivation area [9]. YLD symptoms begin in the middle and lower layers of the tree canopy, with the leaves gradually turning yellow from the tips, and a distinct boundary between the yellow and green areas. The yellowing symptoms spread to the upper leaves, new leaves develop poorly, the canopy size significantly decreases, “bunchy top” symptoms appear, and ultimately, the plant dies [10]. Existing reports indicate that the occurrence of YLD is highly correlated with areca palm velarivirus 1 (APV1) infection in Hainan [7,11]. APV1 is the latest member of the genus *Velarivirus* in the family *Closteroviridae*, with a genome composed of positive-sense single-stranded RNA. The virions are flexuous filaments, approximately 1200–1500 nm in length [12]. Members of the *Closteroviridae* family cannot be transmitted mechanically and primarily rely on insect vectors for transmission. Recent studies have shown that the symptom characteristics exhibited after inoculating areca palm with piercing-sucking insects carrying APV1 are similar to YLD, such as *Ferrisia virgata*, *Pseudococcus* sp., *Aleurocanthus spiniferus* Quaintance, etc. [8]. However, there is currently no known virus vector that transmits APV1. Therefore, we are unable to effectively block the transmission routes of APV1, and the lack of specific and effective chemical agents against the virus has led to the rapid and widespread dissemination of YLD, severely impacting the sustainable development of the areca palm industry.

It is well known that the rhizosphere is a hotspot for microbial activity and diversity [13]. Understand the taxonomic and functional composition differences in the rhizosphere microbiome to establish a well-functioning sustainable ecosystem [14]. It is reported that the rhizosphere microbial community is a crucial factor in maintaining the balance of the host plant’s rhizosphere ecosystem, playing a vital role in the host plant’s resistance to various stress conditions [15,16]. Among them, fungi are one of the highly diverse and active microbial communities in the rhizosphere [17,18]. On one hand, plants can actively shape and select their rhizosphere fungal communities through the secretion of photosynthesis and root exudates [19]. Plant root exudates are a significant source of nutrients for rhizosphere fungi, and through these exudates, the colonization of fungi can be influenced. Simultaneously, root exudates can selectively and actively recruit specific microorganisms, thereby influencing the diversity, composition, and functionality of fungal communities [20,21,22]. On the other hand, rhizosphere fungi can promote plant growth, enhance adaptability to external environments, and resist various pest and disease infestations [23,24]. Rhizosphere fungi can directly participate in processes such as plant nutrient extraction and soil nutrient cycling, serving as a crucial driving force for plant production in the rhizosphere ecosystem. They enhance the resistance of host plants to abiotic and biotic stresses by altering their own community structure and composition, such as in cotton, kiwifruit, and beech [25,26,27]. From this, it can be inferred that there exists a certain dynamic regulatory relationship of mutual benefit and symbiosis between most rhizosphere soil fungi and their host plants.

In recent years, with the development of molecular biological technologies such as high-throughput sequencing, an increasing number of researchers have begun to focus on the relationship between rhizosphere microorganisms and their host plants [28,29]. Current hot research mainly focuses on the impact of abiotic factors on plant cultivation systems, such as planting duration, cultivation methods, water and fertilizer management, temperature, and humidity [13,30,31]. Research on the rhizosphere microbiome of betel nut remains relatively limited. This study employs high-throughput sequencing technology to systematically analyze the compositional differences and diversity characteristics of fungal communities in the rhizosphere of healthy and diseased areca palms. This study aims to thoroughly investigate the impact of YLD on rhizosphere fungal communities and explore potential high-quality biocontrol resources, with the goal of providing a theoretical foundation for the prevention and control of YLD.

## 2. Materials and Methods

### 2.1. Material Sources and Experimental Design

**Figure 1:**
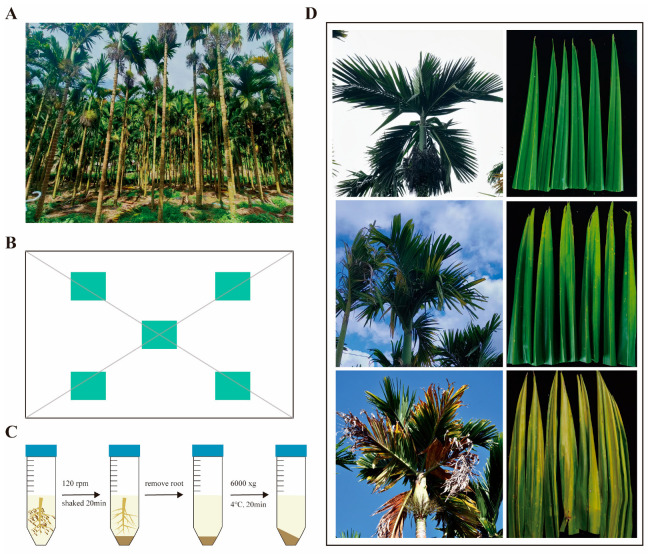
Field identification of symptoms of YLD and sample collection/processing methods. (**A**) Actual field collection site. (**B**) On-site sampling of diseased leaves and soil under the same tree was conducted using the five-point sampling method. (**C**) Pretreatment process of soil samples. (**D**) YLD field identification characteristics.

The test samples were collected from an areca palm plantation (0.5 hectare) affected by YLD in Datang Village, Lingkou Town, Ding’an County, Hainan Province (19°22′ N, 110°17′ E). Leaf samples from approximately 10-year-old Hainan local areca palm varieties, and their corresponding rhizosphere soil samples were collected on 26 July 2023 (the fruit is elliptical in shape with internodes measuring 8 to 15 cm, and it has 6 to 8 effective leaves) (Figure 1A).

Based on the pathological spectrum of YLD in areca palm plantations, soils were collected and categorized into three groups by disease severity: healthy (JK), mildly diseased (MD), and severely diseased (SD). According to the disease grading criteria for YLD established by Tang et al. (2018) [32], JK refers to plants with green leaves numbering five or more, where only the oldest bottom leaf has yellowing or disease spots; MD refers to plants with green leaves numbering four or more; and SD refers to plants with green leaves numbering two or fewer (Figure 1D). Each treatment was conducted with five biological replicates, and three samples were mixed for each replicate. Leaf samples were collected from 15 healthy areca palms, 15 mildly diseased areca palms, and 15 severely diseased areca palms, along with their corresponding rhizosphere soil samples. Rhizosphere soils were collected from under the same trees using the same method.

### 2.2. YLD (APV1) Pathogen Detection

For each treatment, areca palms with consistent growth were randomly selected. For plants exhibiting yellowing symptoms, samples from the junction of the yellow and green areas of the leaflets were collected. For plants without yellowing symptoms, samples from the tips of the leaflets of the second to third last pinnate compound leaves were collected. One sample was collected from each plant, placed in a sterile bag, sealed, and transported to the laboratory under dry ice preservation.

Total RNA was extracted from the areca palm leaves by following the manufacturing instructions of the Polysaccharide Polyphenol Plant RNA Extraction Kit (Coolaber #RE661, Coolaber, Beijing, China). After determining the concentration of RNA samples using the Unano-2000 micro nucleic acid analyzer (Hangzhou Yomim Instrument Co., Ltd., Hangzhou, China), they were stored at −80 °C. Then, 1 μL RNA was taken as the template, 1 μL of OligdT Primer (50 μM) and 1 μL of dNTP Mixture (10 mM) were added, the volume was adjusted to 10 μL with RNase Free dd H_2_O, and then the mixture was reacted at 65 °C for 5 min and placed on ice. Then, a 10 μL mixture, comprising 4 μL 5× Prime Scrip II Buffer, 0.5 μL RNAse inhibitor (40 U/μL), and 1 μL Prime Script II RNase, and 4.5 μL RNase Free dd H_2_O were added to the same PCR tube. The reaction was performed at 42 °C for 45 min, 95 °C for 5 min and placed on ice; then the cDNA was stored at −20 °C.

Using the cDNA as template, CPm-F (5′-TACGCAGTCGCAGTTTCTTC-3′) and CPm-R (5′-CTTGCCCTGCATAGATTCAGTG-3′) were selected to amplify the APV1-CPm region. RT-PCR was performed in a 25 μL reaction system: 12.5 μL of 2× Es Taq MasterMix (Dye) (CWBIO #CW0690M, CWBIO, Beijing, China), 1 μL of each forward and reverse primer, 1 μL of cDNA, and RNase Free dd H_2_O up to 25 μL. Amplification conditions: 94 °C for 5 min, followed by 35 cycles of 94 °C for 30 s, 55 °C for 30 s, and 72 °C for 30 s and finally, 72 °C for 10 min. The PCR product was subjected to gel electrophoresis using 1% agarose, and the results were photographed and observed under UV light.

### 2.3. Collection of Soil Samples from the Rhizosphere of Areca Palm

The sampling tools can be wiped with the original soil from the vicinity of the collection site to minimize contamination as much as possible. Samples were collected using the five-point sampling method. Surface vegetation and other impurities were removed with a shovel. The soil was excavated with a hoe to locate fine roots of trees (diameter ≤ 2 mm), which were then cut using pruning shears. The collected root samples should be portioned into sampling bags, labeled, sealed, and immediately placed in dry ice for preservation, then transported at low temperature to the laboratory for the collection of rhizosphere soil (Figure 1B).

Gently shake off the large clumps of soil from the roots on the clean bench, and weigh approximately 15 g of the root sample. Transfer the mixture to a 250 mL centrifuge tube (contained 200 mL of 1× PBS buffer) and shake it at 120 rpm for 20 min. Use sterile forceps to remove the root system from the centrifuge tube, and centrifuge the remaining suspension at 4000× *g* and 4 °C for 10 min. After discarding the supernatant, the soil samples were stored at −80 °C for further use (Figure 1C).

### 2.4. DNA Extraction and PCR Amplification

For the rhizosphere soil, fungi genome DNA was extracted from 0.5 g soil using a Soil Genomic DNA Purification Spin Kit (Beyotime #D0093S, Beyotime, Shanghai, China). The extracted DNA was quantified and assessed for purity using the NanoDrop One microvolume spectrophotometer (Thermo Fisher Scientific, Waltham, MA, USA), and its quality was evaluated through 1% agarose gel electrophoresis. Using the extracted DNA as template, ITS1F (5′-CTTGGTCATTTAGAGGAAGTAA-3′) and ITS2R (5′-GCTGCGTTCTTCATCGATGC-3′) were selected to target and amplify the ITS1–ITS2 region [16]. A 20 μL amplification reaction system was employed: 4 μL of 5× FastPfu Buffer, 2 μL of dNTPs (2.5 mM), 0.8 μL each of forward and reverse primers (10 μM), 0.4 μL FastPfu DNA Polymerase, 0.2 μL BSA, 1 μL template DNA (10 ng). Add dd H_2_O to bring the mixture to 20 μL. PCR reaction parameters: initial denaturation at 95 °C for 3 min, 27 cycles including denaturation at 95 °C for 30 s, annealing at 55 °C for 30 s, extension at 72 °C for 45 s, final extension at 72 °C for 10 min, and storage at 4 °C. After purification using a Universal DNA Purification and Recovery Kit (TIANGEN #DP214, TIANGEN, Beijing, China), the samples were sent to the company for high-throughput sequencing on the Illumina Novaseq platform.

### 2.5. Data Analysis

After removing the adapter and primer sequences, the raw sequences were quality-filtered using QIIME 1.91 (http://qiime.org) [33]. Assemble the split sequences using FASTP 0.19.3 (https://github.com), and filter out short sequences and low-quality sequences [34]. After classifying the fungal dataset using UNITE v7.2 (https://unite.ut.ee), remove the unmatched ITS sequences from the database [35]. The α diversity analysis (ACE, Chao1, Shannon, and Simpson indices) was conducted using Mothur v1.30.2 (https://www.mothur.org) [36]. Based on the Bray–Curtis algorithm, β diversity was analyzed using PCoA [37]. Gephi 0.10.1 (https://gephi.org) was used to visualize the network [38]. Compare the diversity of fungal populations through analysis of molecular variance (AMOVA).

Statistical analysis was performed using SPSS 22.0 (https://www.ibm.com/analytics/spss-statistics-software, accessed on 12 August 2025). One-way ANOVA and Duncan’s test were employed to assess the diversity of microbial levels (*p* < 0.05).

## 3. Results

### 3.1. YLD (APV1) Pathogen and Soil Sample Testing

**Figure 2:**
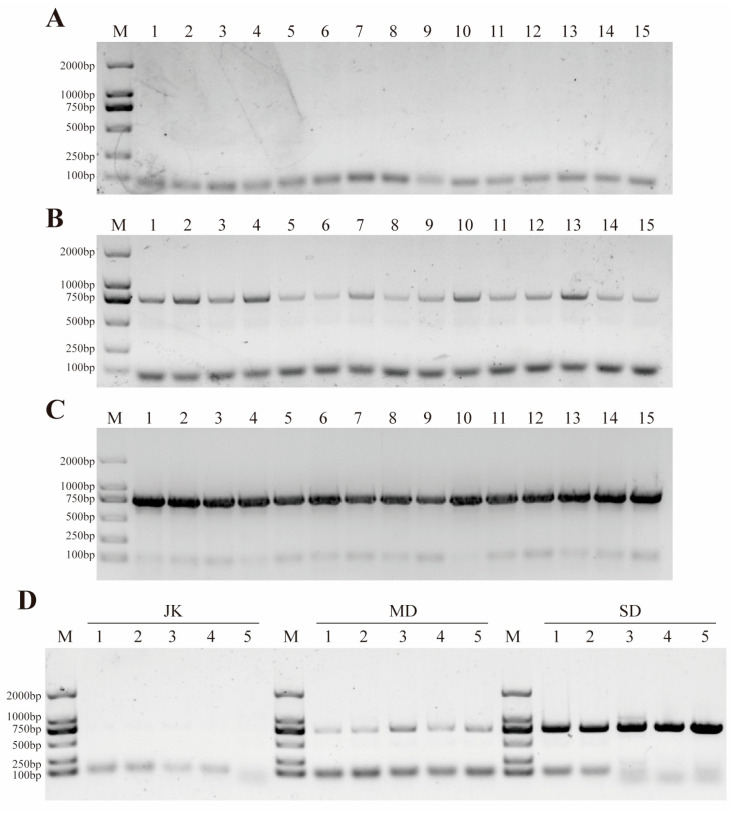
RT-PCR detection of pathogens (APV1) in healthy and diseased areca palms. (**A**) represents healthy areca palm. (**B**) represents a mildly diseased areca palm. (**C**) represents a severely diseased areca palm. (**D**) represents the samples from different groups after mixing.

In this study, a total of 45 leaf samples were collected, including 15 healthy areca palms and 30 diseased areca palms. At the same time, 45 samples of rhizosphere soil corresponding to each tree were collected. To determine whether the symptoms of the diseased leaves were caused by APV1, RT-PCR was used for detection. The results showed that APV1 was detected in all 30 diseased samples (Figure 2B,C). It is noteworthy that the virus could not be detected in any of the 15 healthy samples, even when we pooled every three samples together (Figure 2A,D). In addition, the A260/A280 ratio of the extracted soil fungal genomic DNA ranged between 1.8 and 2.0, and the A260/A230 ratio was greater than 2.0, making it suitable for downstream PCR amplification and high-throughput sequencing analysis (Table S1).

### 3.2. Alpha Diversity Analysis of Sequencing Data

**Figure 3:**
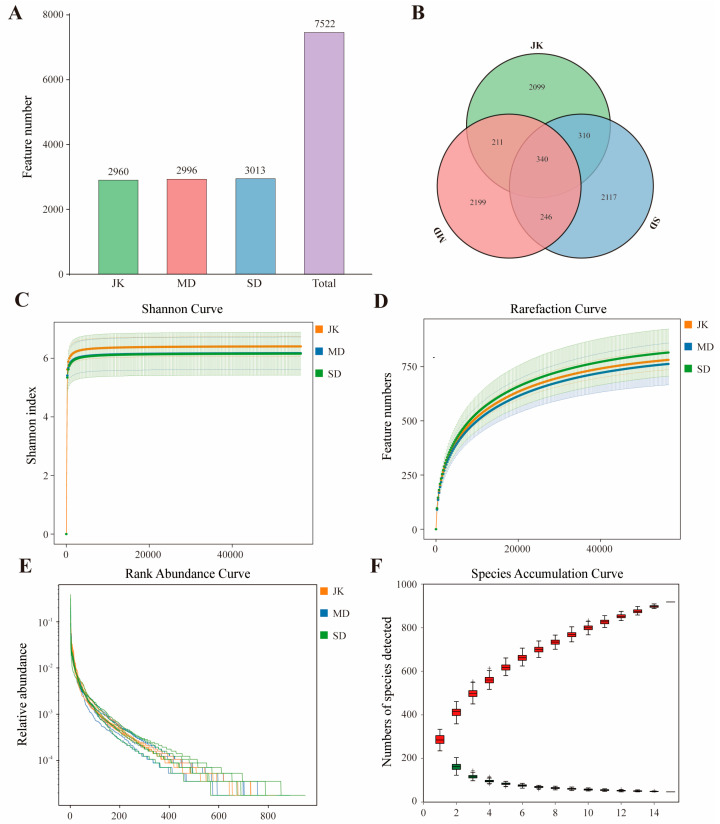
Quality assessment of sequencing data for the rhizosphere fungal community of the areca palm. (**A**) Distribution plot of the number of ASVs among different groups. (**B**) Venn diagram of ASVs among different groups. (**C**) Sample Shannon curve. (**D**) Sample rarefaction curve. (**E**) Sample rank abundance curve. (**F**) Genus-level species accumulation curve.

High-throughput sequencing of 15 samples was performed using the Illumina MiSeq system (the samples included five healthy areca rhizosphere soil fungi (JK1–5) samples, five mildly diseased areca rhizosphere soil fungi (MD1–5) samples, and five severely diseased areca rhizosphere soil fungi (SD1–5) samples). A total of 1,200,251 paired−end raw reads were generated, and 1,020,333 Clean Reads were obtained after quality control and splicing (the distribution of sequence lengths within the corresponding range for each sample is shown in Supplemental Figure S1). The Clean Reads per sample ranged between 66,030 and 70,195 sequences, with an average of 68,022 sequences (Table 1). A total of 7522 ASVs were identified, including 2960 healthy areca palms, 2996 mildly diseased areca palms, and 3013 severely diseased areca palms (Figure 3A) (the ASVs contained in samples with different disease statuses are shown in Figure S2). There were 340 ASVs shared between health and areca palm samples of different disease severities, accounting for 4.52% of the total ASVs (Figure 3B). The rarefaction curve indicates that the sequencing depth has essentially covered all species in the sample (Figure 3D). The Shannon curve tends to smooth out, indicating that the sequencing depth is sufficient to capture the species abundance present in the sample (Figure 3C). The rank abundance curve and species accumulation curve further illustrate the richness and evenness of species contained in the samples (Figure 3E,F). The fungal community coverage index reached over 99%, indicating that the sequencing results with a similarity of 0.03 can accurately reflect the characteristics of the microbial community (Table 1). Therefore, the Alpha diversity index in the sequencing samples can represent the abundance and diversity of fungi in the rhizosphere of the areca palm.

The main Alpha diversity indices include ACE, Chao1, Shannon, and Simpson. The statistical results indicate that (Table S2), compared to healthy samples, the Alpha diversity indices showed a decreasing trend at the initial stage of the disease, with reductions of 15.407, 20.158, 1.178% and 0.231, respectively, and there was no significant difference between the groups. In the late stage of the disease, ACE and Chao1 increased by 39.163 and 33.861, respectively, compared to JK, while Simpson and Shannon decreased by 4.2% and 0.247, respectively, compared to JK. Compared to the initial stage of the disease, the ACE and Chao1 indices increased by 54.57 and 54.019, respectively, in the later stage, while the Simpson and Shannon indices decreased by 3.02% and 0.016, respectively. Overall, with the increase in disease severity, the diversity of fungal communities showed that depletion, species abundance, and richness first decreased and then increased; phylogenetic diversity increased; and the structure of soil fungal communities underwent significant changes.

### 3.3. Composition and Phylogeny of Areca Palm Rhizosphere Fungal Communities

**Figure 4:**
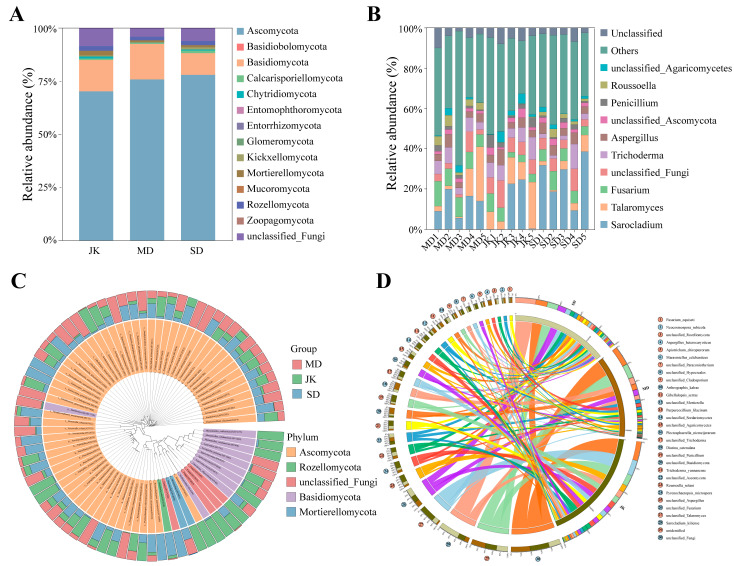
Analysis of fungal species abundance in the rhizosphere of areca palm. (**A**) Bar chart of the abundance of major fungi at the phylum level. (**B**) Bar chart of the major fungal composition at the genus level based on absolute abundance. (**C**) Phylogenetic tree of species, top 80 abundance ratio. (**D**) Genus-level species collinearity plot.

High-throughput sequencing revealed the composition and diversity of fungal communities in different samples. The results of taxonomic annotation (Table S3) indicate that the rhizosphere fungi of areca palms with different disease severity levels belong to 14 phyla, 45 classes, 112 orders, 285 families, 591 genera, and 917 species. The relative abundance of dominant fungi changes significantly with the increase in disease severity across different disease grades. At the phylum level (Figure 4A,C and Figure S4), a total of 14 phyla were detected, among which 6.21% were unclassified phyla. They primarily include *Ascomycota*, *Basidiomycota*, *Rozellomycota*, *Mortierellomycota*, etc., accounting for over 93%. The relative abundances of *Calcarisporiellomycota*, *Chytridiomycota*, *Kickxellomycota*, *Glomeromycota*, *Entorrhizomycota*, *Entomophthoromycota*, *Mucoromycota*, *Mortierellomycota*, *Rozellomycota*, *Zoopagomycota*, and *Zoopagomycota* were all less than 1%. Among them, the abundance of *Ascomycota* was the highest in samples of different health levels, and its relative abundance showed a rapid increasing trend (70.25~77.99%) with the escalation of disease severity. Next is *Basidiomycota* (10.18~16.69%), with its relative abundance showing an initial increase followed by a subsequent decrease (Table S4).

At the genus level (Figure 4B and Table S5), a total of 591 genera were detected. In response to the invasion of APV1 virus, the relative abundance of different genera has undergone varying degrees of change. There are eight genera with relative abundances exceeding 1% across all three groups, including *Sarocladium*, *Talaromyces*, *Fusarium*, *Trichoderma*, *Aspergillus*, *Penicillium*, *Plectosphaerella*, and *Pyrenochaetopsis* (Figure S5 and Table 2). With the increase in disease severity, the relative abundance of *Sarocladium* (9.48~25.58%) showed a rapid upward trend, and there were significant differences among sample groups with varying degrees of disease. The relative abundances of *Trichoderma* (4.2~7.6%), *Penicillium* (1.4~3.3%), and *Talaromyces* (3.75~11.47%) all exhibited a decreasing trend. Conversely, the relative abundances of *Aspergillus* (3.97~4.68%), *Plectosphaerella* (1.36–2.66%), and *Pyrenochaetopsis* (1.21~2.10%) initially decreased and then increased. In addition, the relative abundance of *Fusarium* gradually decreased, with significant differences observed among different disease severity levels, which were 5.19% (JK), 9.02% (MD), and 6.0% (SD), respectively.

### 3.4. Beta Diversity Analysis of Areca Palm Rhizosphere Fungal Communities

**Figure 5:**
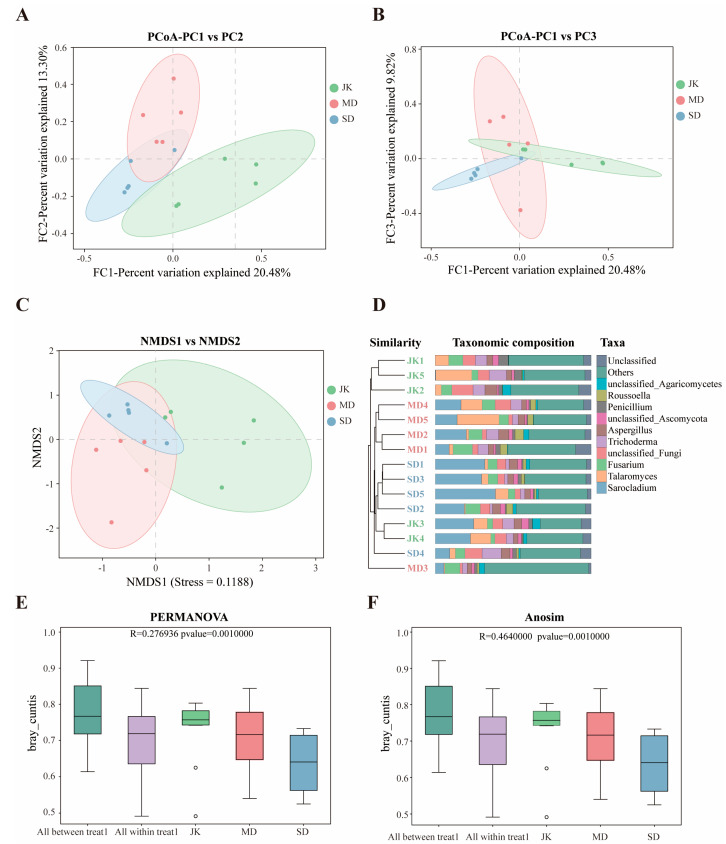
Fungal community structure and cluster analysis based on Bray–Curtis distance. (**A**,**B**) PCoA analysis chart of different disease severity levels. (**C**) NMDS analysis chart. (**D**) Clustered tree bar chart combination. (**E**,**F**) PERMANOVA/Anosim analysis box plots.

To clarify the similarities between healthy betel palm and YLD samples, hierarchical clustering of ASVs was performed based on Bray–Curtis distance. The PCoA results (Figure 5A,B) showed that the first principal coordinate axis accounted for 20.48%, the second principal coordinate axis accounted for 13.30%, and the third principal coordinate axis accounted for 9.82%. The results indicate significant differences in fungal communities between different groups, with samples of the same health level within each group tightly clustered and highly similar. This is highly consistent with the NMDS analysis results (stress < 0.2, indicating the reliability of the NMDS analysis results) (Figure 5C). The UPGMA dendrogram (Figure 5D) shows that at a distance of 0.9, the rhizosphere fungal communities of healthy areca palm (JK), mildly diseased areca palm (MD), and severely diseased areca palm (SD) form independent branches (except for JK3, JK4, SD4, and MD3, which exhibit a high degree of similarity). Similarly to the results of the sample clustering heatmap (Figure S6), this indicates that the degree of disease severity can significantly affect the community composition and structure of the rhizosphere fungi in the areca palm.

To further examine the significant differences among different groups (two or more groups), PERMANOVA/Anosim analysis was employed to assess β-diversity. The R^2^ obtained from PERMANOVA analysis represents the degree to which different groups explain the sample differences (R^2^ = 0.276936). The results indicate that there is a significant degree of variation among the samples across different groups (Figure 5E). The R value obtained from Anosim analysis indicates that the degree of difference between groups is greater compared to the differences within groups (Figure 5F). The smaller the *p*-value, the greater the significance of the differences between groups. A statistically significant *p*-value (*p* = 0.001000) indicates that the grouping is meaningful (Figure 5E,F and Table 3). Overall, the areca rhizosphere fungal samples with different disease severity levels were classified into minor difference groups, and the inter-group differences were significantly greater than the intra-group differences.

### 3.5. Analysis of Dominant Fungal Groups in the Rhizosphere of Areca Palm

**Figure 6:**
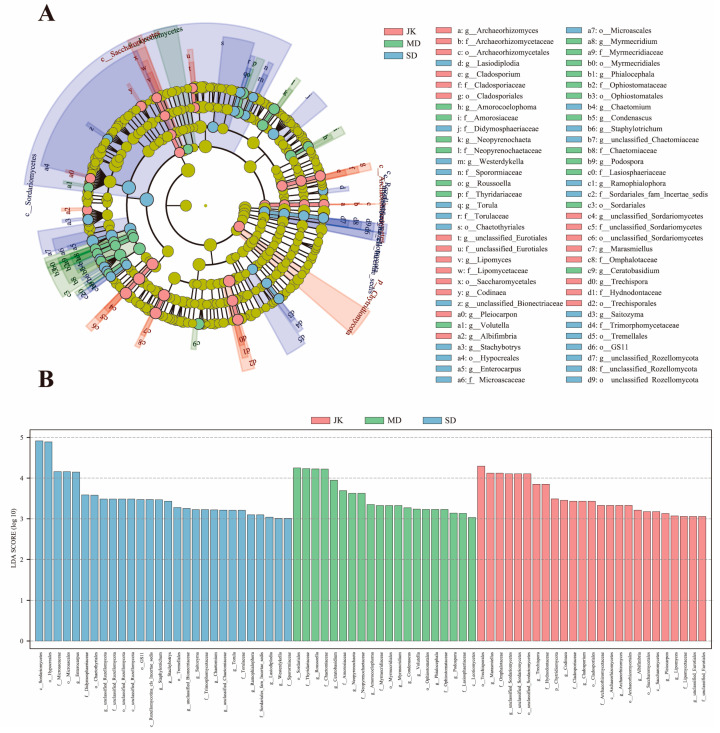
LEfSe analysis of rhizosphere fungal communities in areca palm with different disease severity levels. (**A**) Evolutionary branching diagram. (**B**) LDA statistics.

To confirm the impact of fungal communities on areca palm samples, we conducted LEfSe analysis on those with LDA scores above 3.0. The LEfSe analysis revealed that the rhizosphere fungi of the areca palm with different disease severity levels possess their own unique biomarkers, namely distinct abundant species (Figure 6). The results showed that there were 73 significantly enriched fungal taxa (*p* < 0.05) among the samples across different groups. At the phylum level, *Ascomycota*, as the most abundant taxon, is also a fungus significantly enriched in JK, MD, and SD. At the genus level, the biomarkers of rhizosphere fungi in healthy areca palm (JK) are *Archaeorhizomyces*, *Cladosporium*, *Lipomyces*, *Codinaea*, *Pleiocarpon*, *Albifimbria*, *Marasmiellus*, and *Trechispora*. The biomarkers for rhizosphere fungi in mildly diseased areca palm (MD) are *Amorocoelophoma*, *Neopyrenochaeta*, *Roussoella*, *Volutella*, *Myrmecridium*, *Phialocephala*, *Condenascus*, *Podospora*, and *Ceratobasidium*. The biomarkers for rhizosphere fungi in severely diseased (SD) areca palm are *Lasiodiplodia*, *Westerdykella*, *Torula*, *Stachybotrys*, *Enterocarpus*, *Chaetomium*, *Staphylotrichum*, *Ramophialophora*, and *Saitozyma*. The statistical comparison results of the relative abundance of signature fungi at the genus level are shown in Figure S7.

### 3.6. Network Analysis of Fungal Communities in the Rhizosphere of Areca Palm

**Figure 7:**
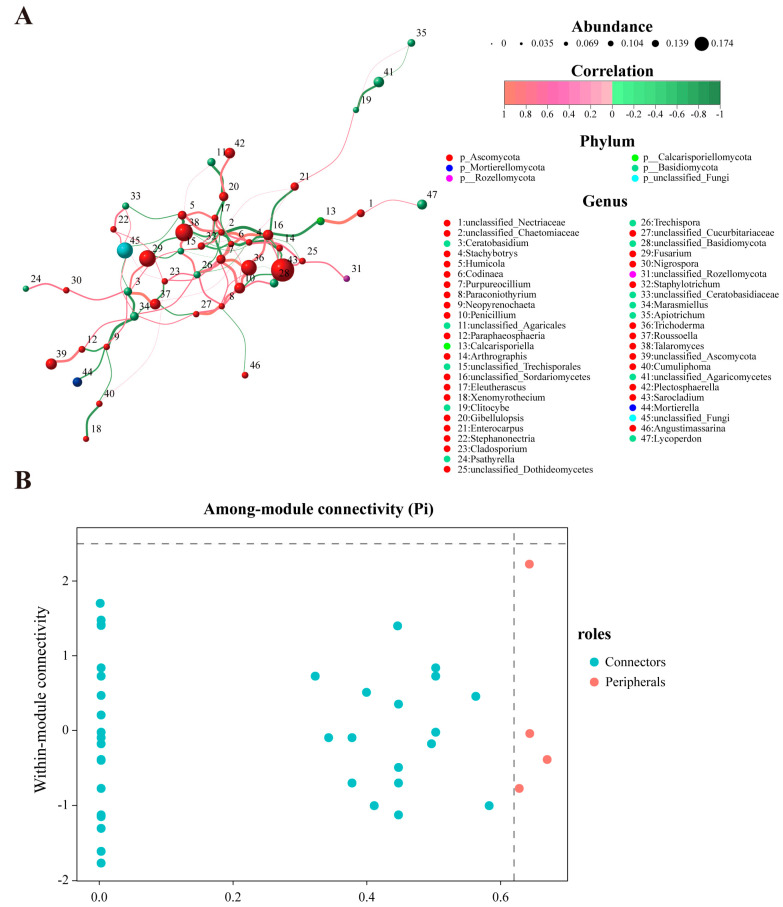
Co-occurrence network of rhizosphere fungal communities in the areca palm with different disease severity levels. (**A**) Network diagram of species at the genus level. Circles represent species, with the size of the circle indicating species abundance; lines denote correlations between two species, with the thickness of the lines representing the strength of the correlation. Red indicating positive correlations, and green indicates negative correlations. (**B**) Node Zi-Pi Distribution Diagram.

**Figure 8:**
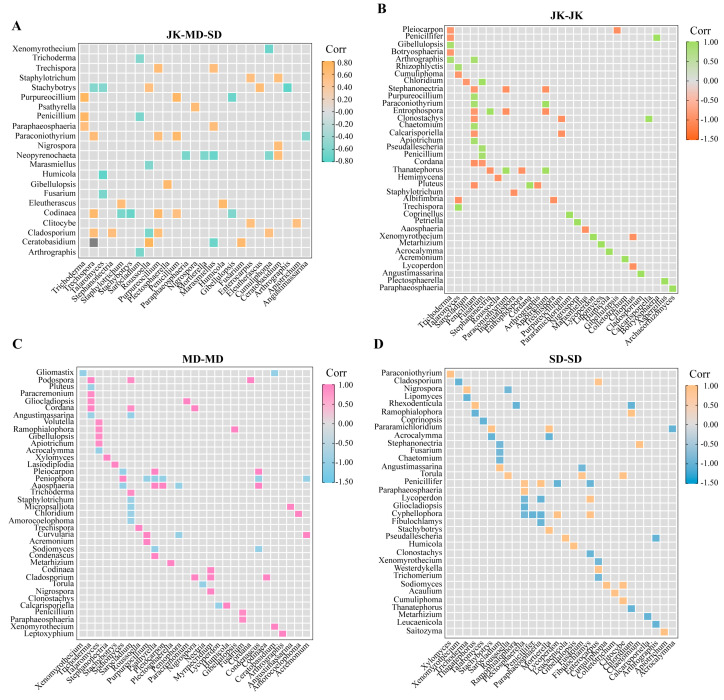
Heatmap of significant differential fungal genus-level interaction forces. (**A**) Interaction forces between different groups. (**B**) The internal interaction forces of healthy areca palms. (**C**) The internal interaction forces in mildly diseased areca palms. (**D**) The internal interaction forces of severely diseased areca palms.

The network analysis assessed the complexity of interactions among fungal genera in the rhizosphere soil of the areca palm under varying degrees of disease incidence. Spearman correlation was used to calculate the correlations among the top 80 fungal genera in the soil. And the co-occurrence networks and topological properties of three different samples were visualized using Gephi (Figure 7A). In the network model, each node represents a microbial genus, and the fungal network is composed of nodes and edges. The results showed that among the 47 fungal genera identified in samples from different groups, 41 negative correlations and 59 positive correlations were detected, distributed between *Ascomycota*, *Basidiomycota*, *Calcarisporiellomycota*, *Mortierellomycota*, and *Rozellomycota*. *Ascomycota* had the highest abundance in the network structure (31 nodes), accounting for 65.98% (Figure 7B). The nodes of *Stachybotrys*, *Codinaea*, *Purpureocillium*, *Arthrographis*, *Eleutherascus*, *Staphylotrichum*, *Trichoderma*, and *Sarocladium* are more numerous; there exist extensive positive and negative interactions, indicating that these groups have closer connections with other groups (Figure 8A).

Additionally, it was found that the rhizosphere soil networks of healthy areca palm and severely diseased areca palm were more complex and compact, with higher centrality and network density and lower modularity (Figure S8). Although the number of fungal genera showing significant differences was similar across different groups, the key species involved in interactions varied. During the relatively stable JK period, *Thanatephorus*, *Pluteus*, *Trichoderma*, *Talaromyces*, *Penicillium*, *Fusarium*, *Apiotrichum* and other microorganisms maintained close connections. Among them, *Thanatephorus* and *Penicillium* exhibited strong interactions (Figure 8B). During the MD period, *Peniophora*, *Trichoderma*, *Psathyrella*, and others dominated the network (Figure 8C). During the SD period, *Fibulochlamys*, *Plectosphaerella*, and *Penicillifer* began to play key roles (Figure 8D). It is worth mentioning that although *Fusarium*, *Humicola*, *Stephanonectria*, etc., did not form the absolute network structure of dominant species, they were still present in the soil at relatively high levels.

### 3.7. Prediction of Biological Functions of Areca Rhizosphere Fungi

**Figure 9:**
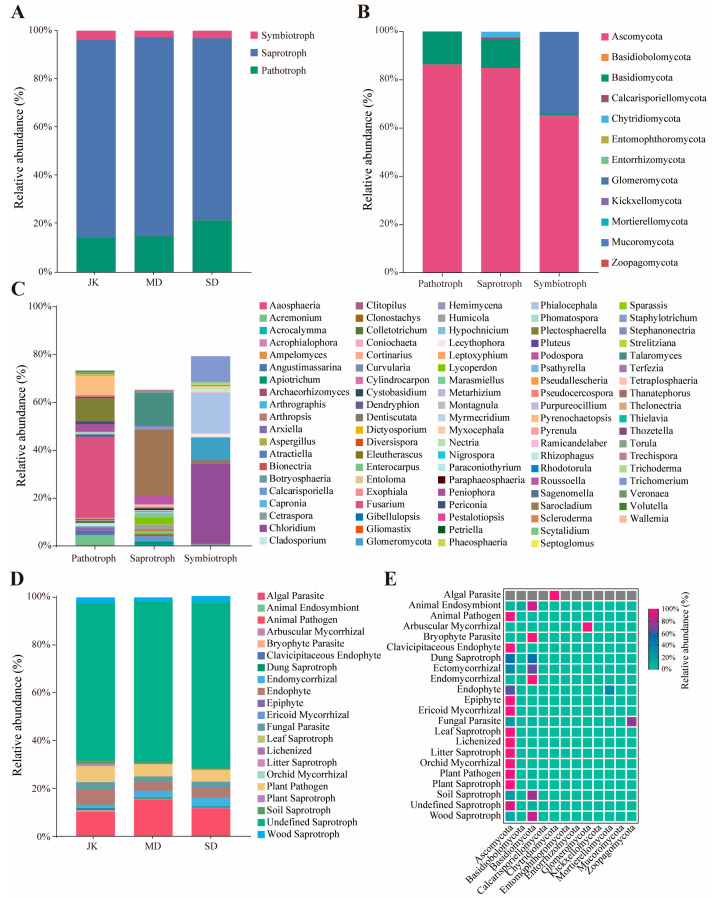
Functional prediction of the rhizosphere fungal community in areca palms with different disease severity levels using FunGuild. (**A**) Statistical analysis of functional groups of rhizosphere fungi based on Trophic Mode for different disease severity levels. (**B**) Fungal species described in (**A**) annotated to the phylum level. (**C**) Fungal genus described in the annotation of (**A**). (**D**) Statistical analysis of functional groups of rhizosphere fungi based on guilds. (**E**) The heatmap of fungi annotated to the phylum level as described in (**D**).

The functional classification of rhizosphere soil fungi and the abundance of samples with different disease severity levels were predicted and analyzed using FUNGuild. According to the statistics on the types of nutrients acquired by microorganisms, the Trophic Mode of the fungal community in the rhizosphere of areca palm is primarily distributed among three functional groups, including Saprotroph, Pathotroph, and Symbiotroph (accounting for 68.74%, 27.78%, and 5.48% of the total abundance of rhizosphere fungi in areca palm, respectively) (Figure 9A). At the phylum level (Figure 9B), these three functional groups all share *Ascomycota* and *Basidiomycota*. *Basidiobolomycota*, *Entomophthoromycota*, *Entorrhizomycota*, *Zoopagomycota,* etc., which are unique components of Pathotroph. *Calcarisporiellomycota*, *Kickxellomycota*, *Mortierellomycota*, *Mucoromycota*, and others primarily rely on Saprotroph for survival. *Glomeromycota* is a phylum of Symbiotroph fungi. At the genus level (Figure 9C), a total of 98 genera were predicted to be involved in the functional classification of fungi, with each genus associated with only one Trophic Mode.

After further dividing the Trophic Mode into guilds, apart from the unknown fungal communities, the rhizosphere fungi of the areca palm were primarily classified into 21 ecological functional groups (Figure 9D). With the increase in the severity of the disease, the relative abundance of Undefined Saprotroph (65.667–69.166%) and Endomycorrhizal (1.492–3.593%) gradually rises. The relative abundances of Plant Saprotroph (0.061–1.012%), Soil Saprotroph (0.822–1.454%), and Bryophyte Parasite (0.018–0.232%) are higher in healthy areca palms. The relative abundance of Endomycorrhizal in severely diseased areca palms is significantly higher than that in healthy areca palms. Furthermore, in the SD region, there are some unique low-abundance ecological functional groups, such as Clavicipitaceous Endophyte and Epiphyte, which may have significant impacts on the biological functions of fungal communities (Figure 9E). Further analysis of the fungal genera constituting the guild revealed that the fungal members composed of Animal Pathogen (14 genera), Plant Pathogen (26 genera), Undefined Saprotroph (37 genera), Dung Saprotroph (10 genera), Endophyte (10 genera), etc., are more numerous and their biological functions are relatively complex. While Bryophyte Parasite (1 genus), Clavicipitaceous Endophyte (1 genus), Animal Endosymbiont (2 genera), Epiphyte (1 genus), and Ericoid Mycorrhizal (1 genus) each contain only one or two genera, they often perform singular biological functions. In addition, genera such as Algal Parasite, Endomycorrhizal, and Plant Saprotroph did not have predicted corresponding biological functions. The specific details are as described in Figure S9.

## 4. Discussion

It is well known that the rhizosphere soil is a highly complex and dynamic ecosystem [37]. This study employed amplicon sequencing to compare the differences in rhizosphere soil fungi between healthy and diseased areca palms, including aspects such as abundance, diversity, structure, composition, and interactions. The results indicate that there are significant differences in the structure of fungal communities and microbial diversity across varying degrees of disease severity. The core fungal communities showed significant variations among healthy areca palms (JK), mildly diseased areca palms (MD), and severely diseased areca palms (SD). Therefore, during the process of areca palm being infected by yellowing disease to varying degrees, the composition and diversity of the rhizosphere fungal community are dynamic. This is consistent with the findings of previous studies, indicating that the dynamic system of the rhizosphere soil environment and microbial community composition is one of the critical factors regulating plant health levels [39,40]. However, there have been no reports on the microbial diversity in the rhizosphere soil of areca palm yellowing disease.

The diversity of soil microbial communities is crucial for maintaining plant growth and development as well as soil health [13]. Fungi are a crucial component of soil biomass, playing a pivotal role in nutrient cycling and biological interactions [41]. The unique ASVs of areca palm plants with varying degrees of disease incidence revealed significant differences in the rhizosphere soil samples of JK, MD, and SD. With the increase in disease severity, the richness and diversity of rhizosphere samples at different disease levels showed a trend of first decreasing and then increasing (with JK > SD), indicating that the severity of yellowing disease affects fungal diversity. Previous studies have shown that the microbial diversity in rhizosphere soil is positively correlated with plant health [42]. This is consistent with our findings that the diversity of rhizosphere fungi in healthy samples tends to be higher than in diseased samples. This study demonstrates that the alpha diversity of areca rhizosphere samples shows no significant differences, whereas the beta diversity exhibits significant variations. This indicates that environmental heterogeneity does not affect fungal diversity, but it does influence the structure and composition of fungal communities. From the perspective of the functional classification predicted by FUNGuild, the fungal nutrient type structures of the samples from the rhizosphere soil of areca palm with three different disease severities were highly similar, with saprophytic fungi being the absolutely dominant type; this is highly consistent with the findings of Ma et al. (2021) [37]. The dominant ecological functional groups in the rhizosphere samples of areca palm are primarily composed of types such as Undefined Saprotroph, Animal Pathogen, and Plant Pathogen, among which *Sarocladium*, *Hypocreales*, *Fusarium*, *Talaromyces*, *Eurotiales*, *Roussoella*, and *Plectosphaerella* are present in all soil samples, exhibiting the highest relative abundance. It is noteworthy that once the environment becomes suitable, *Fusarium*, as a significant plant pathogen and mycotoxin producer, can lead to the occurrence of areca palm yellowing disease, severely affecting agricultural development and economic levels [43,44]. In addition, the necrotrophic fungus *Plectosphaerella* is a destructive plant pathogen that has been proven to cause a variety of plant diseases and pests, such as root rot [45], wilt [46,47], sudden death [48], leaf spot [49], gray mold [50], potato cyst nematode [51], etc. As for whether it will cause areca palm yellowing disease, further verification is needed. Therefore, yellowing disease may lead to a decrease in the resistance of rhizosphere fungi in areca palm to the soil microenvironment and external factors, thereby limiting the sustainable development of the soil.

Microbial ecological networks are commonly used to study interactions among microorganisms, aiding in understanding the responses of microbial communities to external disturbances [52,53]. The key microbial ASVs group screened based on positive and negative interactions can promote soil nutrient cycling, plant growth and development, and protect plants from pathogen infection [54]. Our ecological network analysis results revealed unique patterns of the areca palm rhizosphere soil microbial community under different conditions of yellow leaf disease. The dominance of *Stachybotrys*, *Arthrographis*, *Codinaea*, *Paraconiothyrium*, *Sarocladium*, *Talaromyces*, *Roussoella*, and others in the fungal network indicates that these groups may play a significant role in the development of areca palm yellowing disease. In particular, *Stachybotrys* and *Roussoella* exhibit extensive negative interactions with other genera, but there is a positive interaction between them. This may be aimed at balancing or limiting other microorganisms, thereby contributing to the construction of a more stable fungal community network structure in the rhizosphere soil and enhancing the resistance of rhizosphere microorganisms to external disturbances [37,42]. The abundance of *Trechispora* in the healthy areca palm sample (JK) is absolutely higher than in other disease stages. It is noteworthy that Oliveira et al. (2024) found *Trechispora* to be crucial for the decomposition of organic matter and nutrient cycling [55].

Recent reports suggest that plant rhizospheres may combat stress by recruiting beneficial microorganisms [56]. Through LEfSe analysis, 26 significantly different fungal genera were identified. Among them, 11 are beneficial fungi for plants, and 15 are harmful fungi. *Marasmiellus* is the core dominant genus in healthy areca palms (JK). Previous studies have shown that *Marasmiellus* degrades most plant leaves and woody debris [57]. This indicates that *Marasmiellus* provides the necessary nutrients for the growth of healthy areca palms by decomposing soil organic matter during their growth stages. *Roussoella* is the dominant genus during the mild onset of areca palm yellowing disease (MD). Numerous structurally novel and biologically active secondary metabolites discovered from the strains exhibit potent antiviral, antiparasitic, and immunomodulatory effects [58]. *Saitozyma* is the dominant genus during the severe outbreak of areca palm yellowing disease (SD). The phytase produced by *Saitozyma* strains has been proven to decompose inorganic phosphate and phytic acid (PA) into organic phosphorus and ATP [59]. Under the extremely nutrient-deprived soil conditions in the late stages of disease, *Saitozyma* releases organic phosphorus to provide the nutrients and energy required for the growth of areca palms. Therefore, some beneficial species should be considered as key fungi present in the rhizosphere soil of the areca palm and may play a crucial role in balancing the stability of the fungal interaction network.

## 5. Conclusions

This study is the first to employ amplicon sequencing to analyze the community composition and microbial diversity of rhizosphere fungi in areca palms under different disease severity levels of APV1. Research indicates that the diversity of rhizosphere fungi is positively correlated with plant health status. With the increase in the severity of the disease, the number of harmful fungi in the soil rises, thereby limiting the sustainable development of the soil. However, some potential plant-beneficial microbial communities can serve as key taxa in the areca rhizosphere network, thereby reducing and inhibiting the occurrence of yellowing disease. These findings can provide a deeper understanding of the composition and diversity of the fungal community in the areca palm; at the same time, they also offer new directions for the green control of yellowing disease.

## Figures and Tables

**Table 1:** Genomic DNA quality parameters of rhizosphere microorganisms in areca palm.

Sample	Raw Reads	Clean Reads	Denoised Reads	Merged Reads	Non-Chimeric Reads
JK1	79,876	66,661	66,493	64,119	56,765
JK2	80,159	69,326	69,228	66,782	62,137
JK5	80,096	66,030	65,940	63,685	59,494
JK3	80,137	68,709	68,491	66,223	64,130
JK4	80,100	69,773	69,644	67,332	63,921
SD1	79,653	68,085	68,009	66,196	64,370
SD2	79,992	67,742	67,582	64,911	63,202
SD4	80,026	66,139	66,027	63,489	60,963
SD5	79,984	66,581	66,467	64,344	62,781
SD3	80,254	68,054	67,945	65,956	64,623
MD1	79,896	66,791	66,647	64,294	62,020
MD2	79,967	68,269	67,999	66,118	64,413
MD3	80,149	68,381	68,308	66,737	65,714
MD4	79,969	69,597	69,448	67,934	62,158
MD5	79,993	70,195	70,046	68,079	65,045

**Table 2:** ANOVA of genus-level groups with species abundance exceeding 1%.

Genus	SS	Df	MS	F	P (1:2:3)	P (1:2)	P (1:3)	P (2:3)
*Sarocladium*	0.072	2	0.036	3.227	0.076	0.612	0.033	0.083
*Talaromyces*	0.0160	2	0.008	1.230	0.326	0.634	0.150	0.315
*Fusarium*	0.004	2	0.002	3.566	0.061	0.023	0.426	0.099
*Trichoderma*	0.003	2	0.001	1.428	0.278	0.354	0.118	0.485
*Aspergillus*	0	2	0	0.306	0.742	0.551	0.911	0.481
*Penicillium*	0.001	2	0.001	2.743	0.104	0.078	0.056	0.850
*Plectosphaerella*	0	2	0	0.211	0.813	0.537	0.672	0.844
*Pyrenochaetopsis*	0	2	0	0.616	0.557	0.290	0.546	0.636

Note: Df denotes degrees of freedom; SS stands for sum of squares; MS indicates mean squares; F represents the F-test value; “1” represents JK, “2” represents MD, “3” represents SD.

**Table 3:** PERMANOVA/Anosim statistical tests.

Diffs	Df	SS	MS	F	PERMANOVA			ANOS		
					R^2^	*p*-Value	Psignf	R	*p*-Value	Psignf
treat1	2	1.122	0.561	2.298	0.277	0.001	**	0.464	0.001	*

Note: diffs represent the comparison groups of differences; R^2^ represents the degree to which different groups explain the variation in samples, that is, the ratio of group variance to total variance; R represents the degree of difference; Psignf denotes the significance symbol of *p*-value (*: <0.05, **: <0.001).

## Data Availability

The raw sequencing data presented in this study have been deposited in the NCBI Sequence Read Archive database (registration number: PRJNA1336500). For further inquiries, please directly contact the corresponding authors.

## Supplementary Materials

The following supporting information can be downloaded at https://www.mdpi.com/article/10.3390/jof11110803/s1. Table S1: Genomic DNA quality parameters of rhizosphere microorganisms in areca palm; Table S2: Diversity of rhizosphere fungi in areca palm with different disease severity levels; Table S3: Species annotation table for each grade of samples; Table S4: Phylum-level species abundance table; Table S5: Genus-level species abundance statistical table; Figure S1: The sequence length distribution plot of each sample; Figure S2: Graph of ASVs contained in different samples; Figure S3: Alpha diversity difference index; Figure S4: The main fungal components at the phylum level with relatively high abundance; Figure S5: The dominant fungal components at the genus level with absolute abundance; Figure S6: Heatmap of sample clustering; Figure S7: Statistical comparison results of the relative abundance of signature fungi at the genus level; Figure S8: Correlation network of fungal communities across different samples; Figure S9: Genus-level biological function prediction.
