# Supplementary material for: Comparison of Rhizosphere Fungal Community Changes in Healthy and Yellowing-Leaf-Disease-Affected Areca Palms by High-Throughput Sequencing Technology
Source: J Fungi (Basel). 2025 Nov 12;11(11):803. doi: 10.3390/jof11110803 (PMC12653923; doi:10.3390/jof11110803)
Supplement: Supplementary file 1 [file jof-11-00803-s001.zip › jof-3923741-supplementary.pdf]

Additional information

Table S1. Genomic DNA quality parameters of rhizosphere microorganisms in areca palm.

Sample	DNA concentration (μg/mL)	A260/A280	A260/A230
JK1	72.2	1.83	2.158
JK2	122.55	1.846	2.156
JK3	96.25	1.835	2.158
JK4	50.2	1.803	2.178
JK5	126.85	1.841	2.311
MD1	233	1.857	2.142
MD2	89.15	1.848	2.301
MD3	64.6	1.853	2.221
MD4	116.65	1.872	2.308
MD5	89.7	1.855	2.076
SD1	100.8	1.844	2.265
SD2	94.5	1.831	2.383
SD3	118.15	1.856	2.244
SD4	171.55	1.829	2.195
SD5	145.75	1.865	2.167

Table S2. Diversity of rhizosphere fungi in areca palm with different disease severity levels.

Sample	Feature	ACE	Chao1	Simpson	Shannon	PDwholetree	Coverage
JK1	754	831.2799	806.678	0.9812	6.8447	175.9203	0.998
JK2	780	842.67	831.5	0.976	6.6404	173.041	0.9982
JK3	816	863.263	842.9137	0.9314	6.0832	195.6786	0.9985
JK4	834	895.7219	884.5909	0.9277	6.0911	201.3359	0.9981
JK5	723	757.973	744.9252	0.9687	6.365	180.7	0.9988
MD1	889	975.3534	952.1511	0.9729	6.7921	190.3446	0.9977
MD2	819	896.4773	870.0226	0.9479	6.2451	194.6478	0.9979
MD3	659	709.1987	689.8496	0.9438	5.9429	158.4345	0.9985
MD4	769	805.2768	793.1262	0.9593	6.5281	173.0058	0.9987
MD5	677	727.5651	704.6667	0.9022	5.3609	150.1773	0.9985
SD1	663	727.0212	705.3273	0.8905	5.5628	155.4392	0.9983
SD2	885	937.7948	919.2353	0.9574	6.7577	211.4951	0.9983
SD3	795	875.8001	856	0.9055	5.9909	182.6601	0.9978
SD4	949	1,003.62	979.2739	0.9781	7.07	208.8095	0.9983
SD5	785	842.4842	820.0769	0.8434	5.4057	174.2267	0.9983

Table S3. Species annotation table for each grade of samples.

Sample	Kindom	Phylum	Class	Order	Family	Genus	Species
JK1	1	11	27	76	145	231	265
JK2	1	11	32	75	145	240	284
JK3	1	11	30	73	147	231	283
JK4	1	10	33	77	151	258	309
JK5	1	9	29	71	137	228	270
MD1	1	10	27	74	156	256	315
MD2	1	10	28	74	151	241	292

MD3	1	10	25	62	129	209	234
MD4	1	7	26	63	146	231	277
MD5	1	10	26	65	133	216	266
SD1	1	10	28	71	136	228	265
SD2	1	11	31	77	161	261	302
SD3	1	12	30	73	154	264	306
SD4	1	12	34	80	163	281	333
SD5	1	9	28	73	156	259	302
Total	1	14	45	112	285	591	917

Table S4. Phylum-level species abundance table.

Phylum	JK	MD	SD	Average
Ascomycota	0.7025±0.00923	0.7586±0.01593	0.7799±0.01380	0.7470±0.01063
Basidiomycota	0.1471±0.00923	0.1669±0.01928	0.1018±0.00721	0.1386±0.00888
unclassified_Fungi	0.0847±0.00651	0.0407±0.00785	0.0609±0.00659	0.0621±0.00585
Rozellomycota	0.0226±0.00401	0.0170±0.00298	0.0195±0.00295	0.0197±0.00074
Mortierellomycota	0.0214±0.00388	0.0084±0.00188	0.0108±0.00113	0.0135±0.00184
Calcarisporiellomycota	0.0066±0.00135	0.0037±0.00132	0.0119±0.00174	0.0074±0.00110
Chytridiomycota	0.0085±0.00122	0.0013±0.00031	0.0047±0.00111	0.0048±0.00095
Kickxellomycota	0.0021±0.00035	0.0020±0.00043	0.0081±0.00257	0.0040±0.00093
Glomeromycota	0.0044±0.00055	0.0013±0.00029	0.0022±0.00030	0.0026±0.00043
Entorrhizomycota	1.8E-05±8.9E-06	1.8E-05±0.00923	0.0001±3.9E-05	4.6E-05±1.3E-05
Others	5.7E-05±1.1E-05	1.1E-05±0.00923	8.5E-05±2.1E-05	5.1E-05±1.0E-05

Table S5. Genus-level species abundance statistical table.

Genus	JK	MD	SD	Average
Sarocladium	0.0948±0.03251	0.1296±0.01456	0.2558±0.02924	0.1601±0.02254
Talaromyces	0.1147±0.01843	0.0903±0.02852	0.0375±0.00718	0.0808±0.01049
Fusarium	0.0519±0.00679	0.0902±0.00581	0.0640±0.00475	0.0687±0.00520
unclassified_Fungi	0.0846±0.00727	0.0407±0.00878	0.0609±0.00737	0.0621±0.00585
Trichoderma	0.0760±0.00592	0.0566±0.00527	0.0420±0.01149	0.0582±0.00454
Unclassified	0.0568±0.00396	0.0468±0.00806	0.0386±0.00436	0.0474±0.00243
Aspergillus	0.0457±0.00489	0.0397±0.00392	0.0468±0.00259	0.0440±0.00102
unclassified_Ascomycota	0.0263±0.00367	0.0175±0.00104	0.0243±0.00157	0.0227±0.00122
Penicillium	0.0330±0.00544	0.0157±0.00221	0.0140±0.00200	0.0209±0.00280
Roussoella	0.0024±0.00029	0.0365±0.00384	0.0220±0.00290	0.0203±0.00455
unclassified_Agaricomycetes	0.0276±0.00568	0.0170±0.00411	0.0135±0.00230	0.0194±0.00196
Plectosphaerella	0.0266±0.01244	0.0136±0.00444	0.0178±0.00486	0.0193±0.00176
unclassified_Sordariomycetes	0.0301±0.00487	0.0195±0.00217	0.0071±0.00066	0.0189±0.00307
Lycoperdon	0.0013±0.00023	0.0472±0.02089	0.0020±0.00011	0.0168±0.00700
Pyrenochaetopsis	0.0230±0.00474	0.0121±0.00172	0.0160±0.00228	0.0164±0.00118
unclassified_Hypocreales	0.0111±0.00162	0.0198±0.00317	0.0141±0.00210	0.0150±0.00118
Mortierella	0.0213±0.00431	0.0083±0.00212	0.0108±0.00127	0.0134±0.00183
Gibellulopsis	0.0094±0.00131	0.0161±0.00424	0.0124±0.00124	0.0126±0.00090
Purpureocillium	0.0181±0.00280	0.0089±0.00136	0.0095±0.00147	0.0121±0.00137
Humicola	0.0014±0.00029	0.0220±0.01103	0.0084±0.00280	0.0106±0.00278
Marasmiellus	0.0260±0.00886	2E-05±0.00001	0.0048±0.00244	0.0103±0.00368
unclassified_Basidiomycota	0.0094±0.00165	0.0149±0.00433	0.0047±0.00070	0.0097±0.00136

Acremonium	0.0026±0.00028	0.0166±0.00182	0.0079±0.00126	0.0091±0.00189
Ceratobasidium	1E-05±0.00001	0.0172±0.00211	0.0082±0.00185	0.0085±0.00229
unclassified_Agaricales	0.0036±0.00079	0.0171±0.00418	0.0045±0.00143	0.0084±0.00199
Enterocarpus	0.0001±0.00007	1E-05±0.00000	0.0252±0.00580	0.0084±0.00385
Calcarisporiella	0.0066±0.00151	0.0037±0.00147	0.0119±0.00195	0.0074±0.00110
Trechispora	0.0133±0.00347	0.0007±0.00034	0.0008±0.00032	0.0049±0.00193
Eleutherascus	5E-05±0.00001	0.0111±0.00625	0.0003±0.00005	0.0038±0.00169
Others	0.1865±0.01163	0.2067±0.01353	0.2361±0.01805	0.2098±0.00664

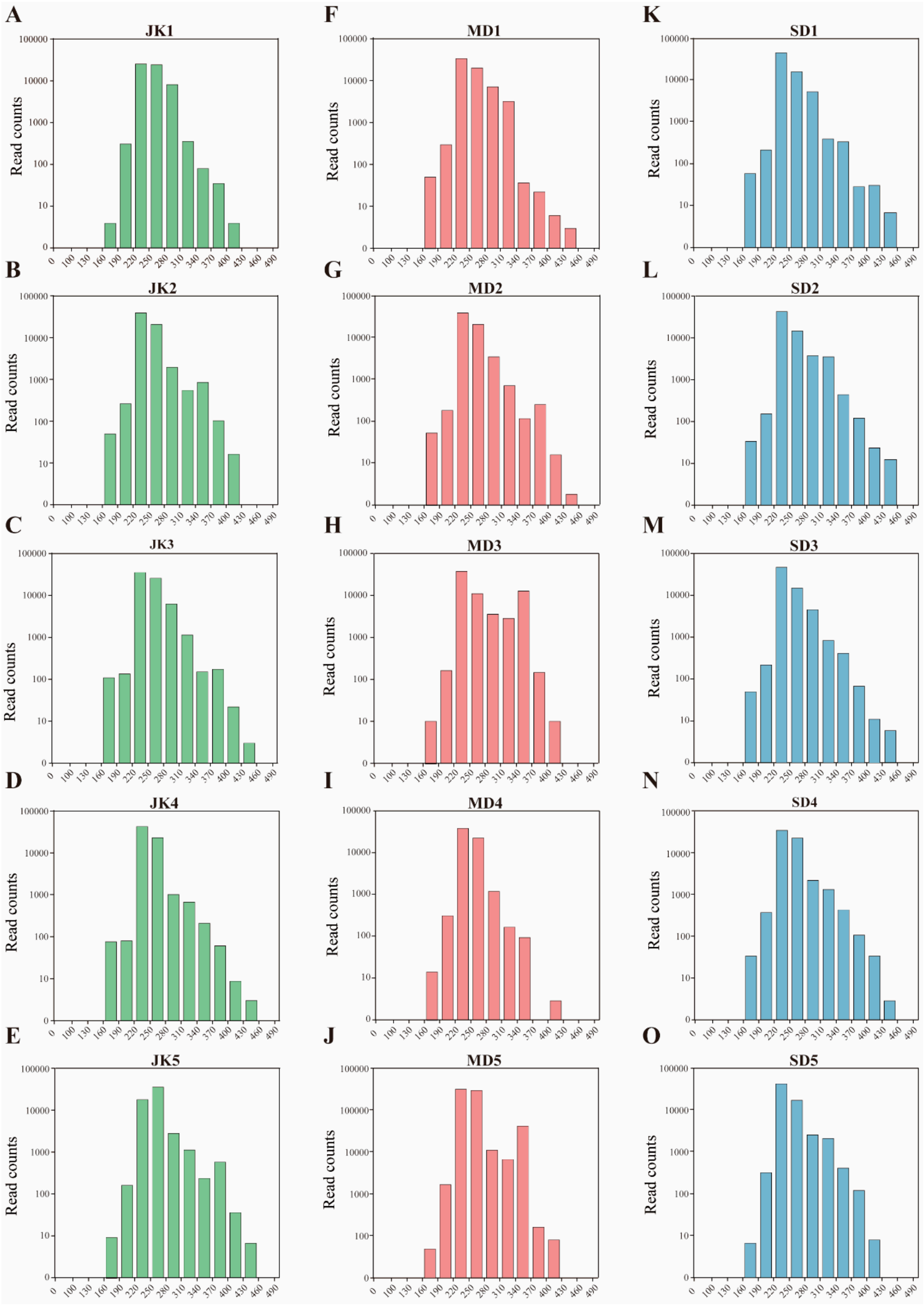

Figure S1. The sequence length distribution plot of each sample. JK represents healthy areca palm, MD represents mildly diseased areca palm, and SD represents severely diseased areca palm.

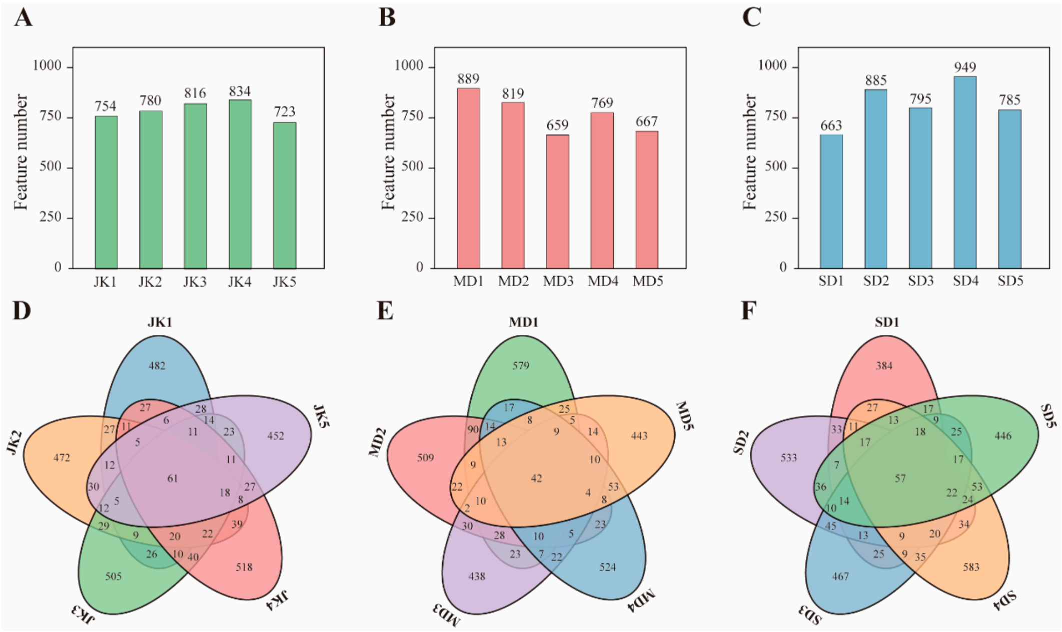

Figure S2. Graph of ASVs contained in different samples. (A), (B), and (C) represent the distribution of ASVs counts in different samples. (D), (E) and (F) are Venn diagrams of ASVs in different samples.

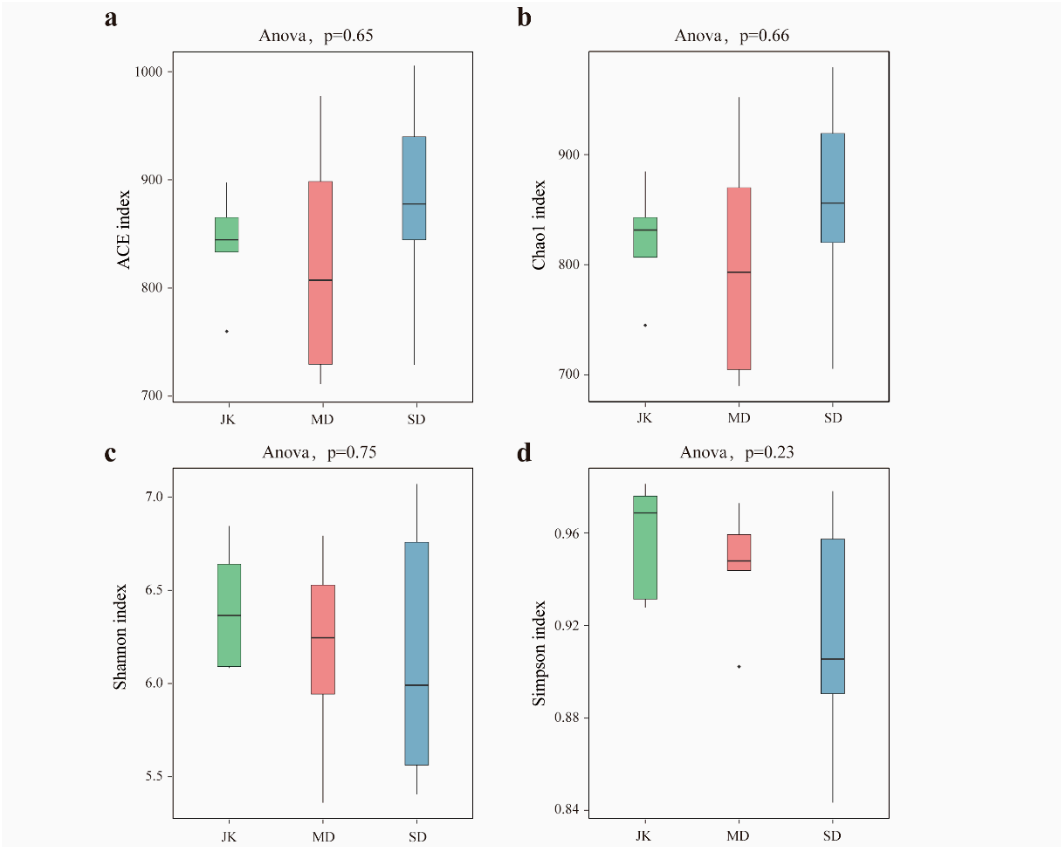

Figure S3. Alpha diversity difference index. (A) ACE index. (B) Chao1 index. (C) Shannon index. (D) Simpson index.

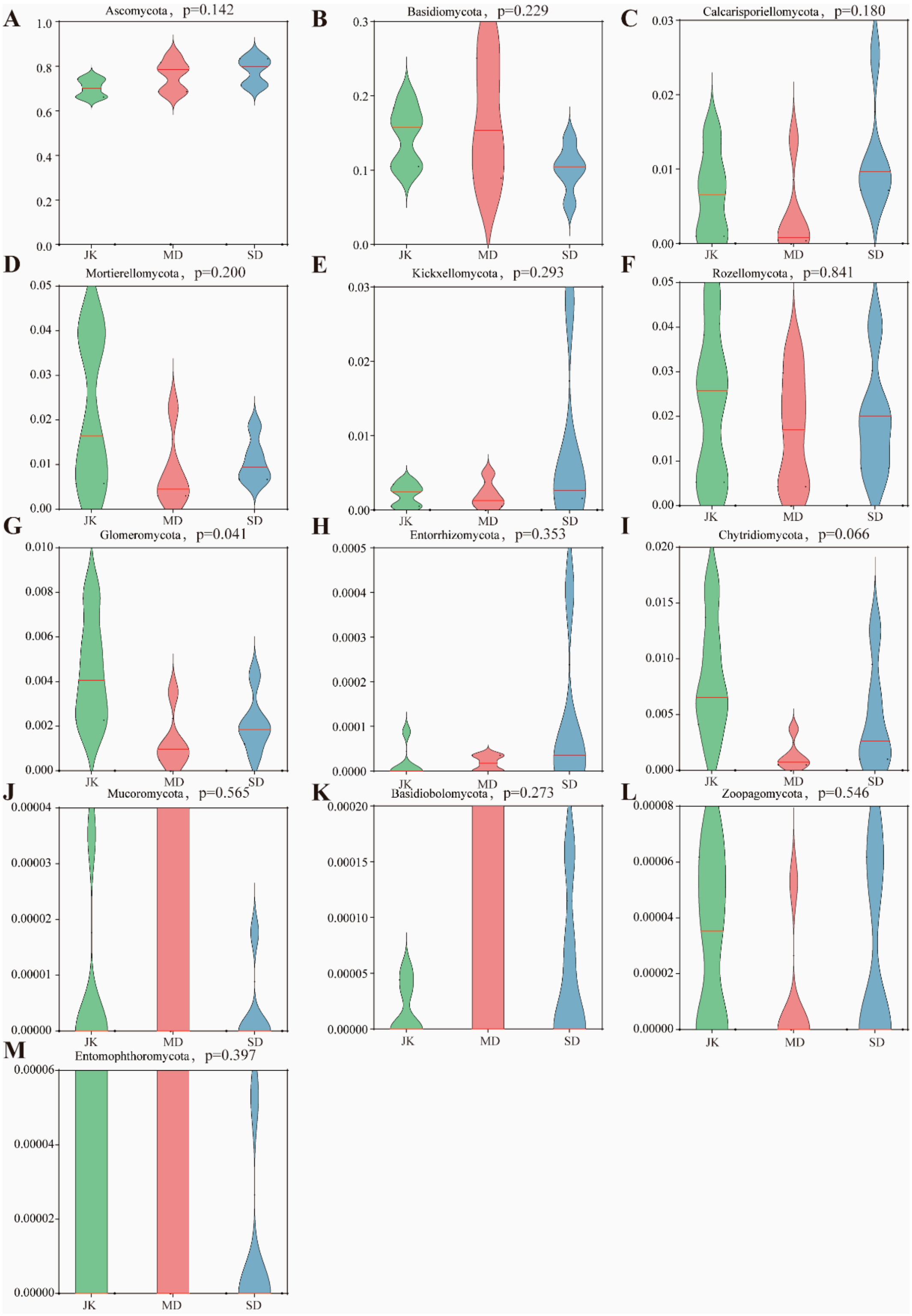

Figure S4. The main fungal components at the phylum level with relatively high abundance. (A)-(M) Phylum-level violin plots representing the absolute abundance of fungi across different disease severity levels.

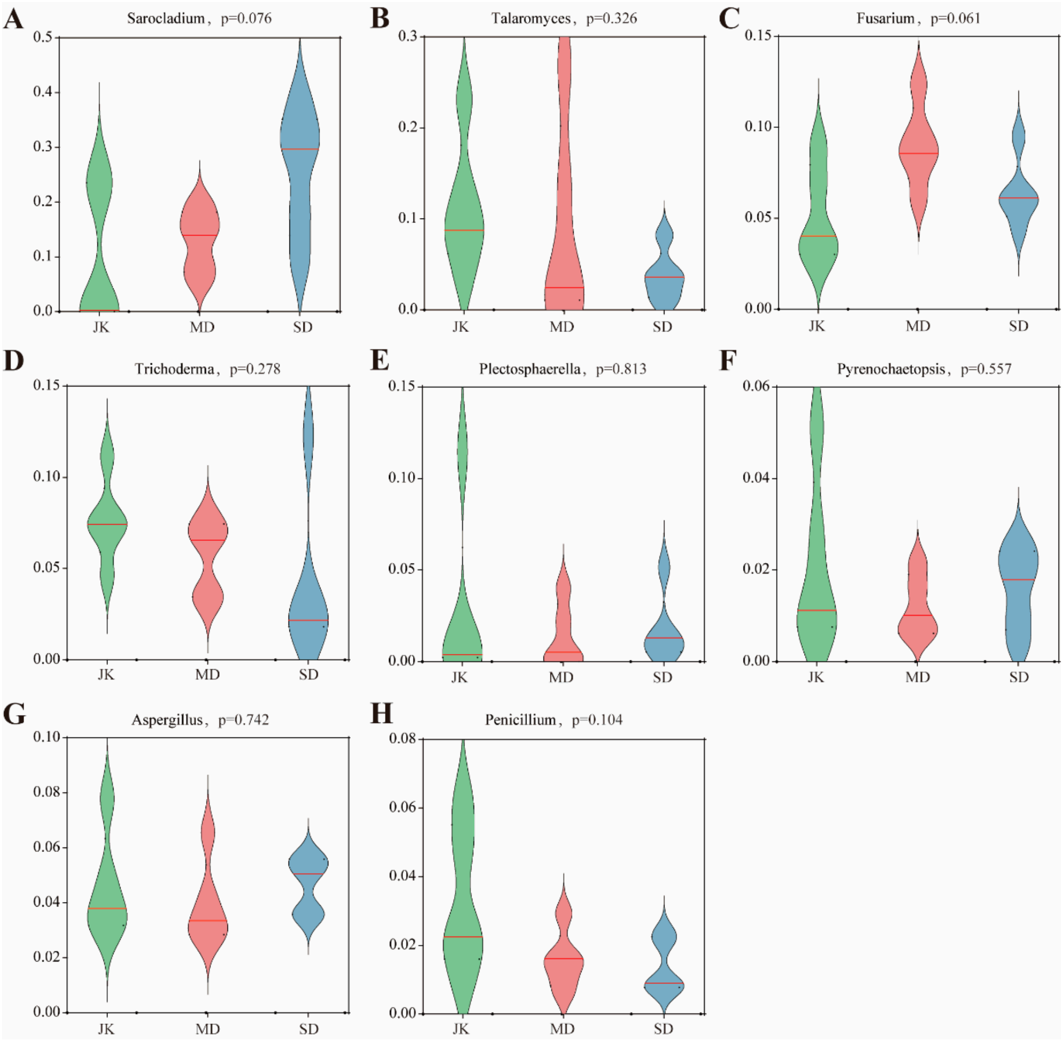

Figure S5. The dominant fungal components at the genus level with absolute abundance. (A)-(H) Violin plots at the genus level, with intergroup abundance exceeding 1%.

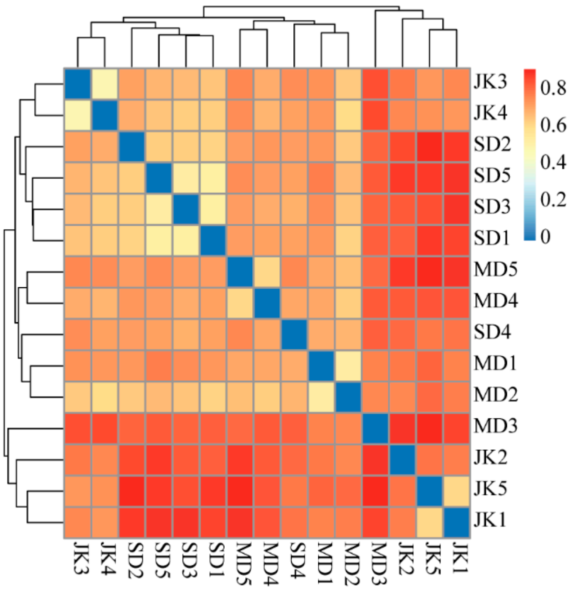

Figure S6. Heatmap of sample clustering.

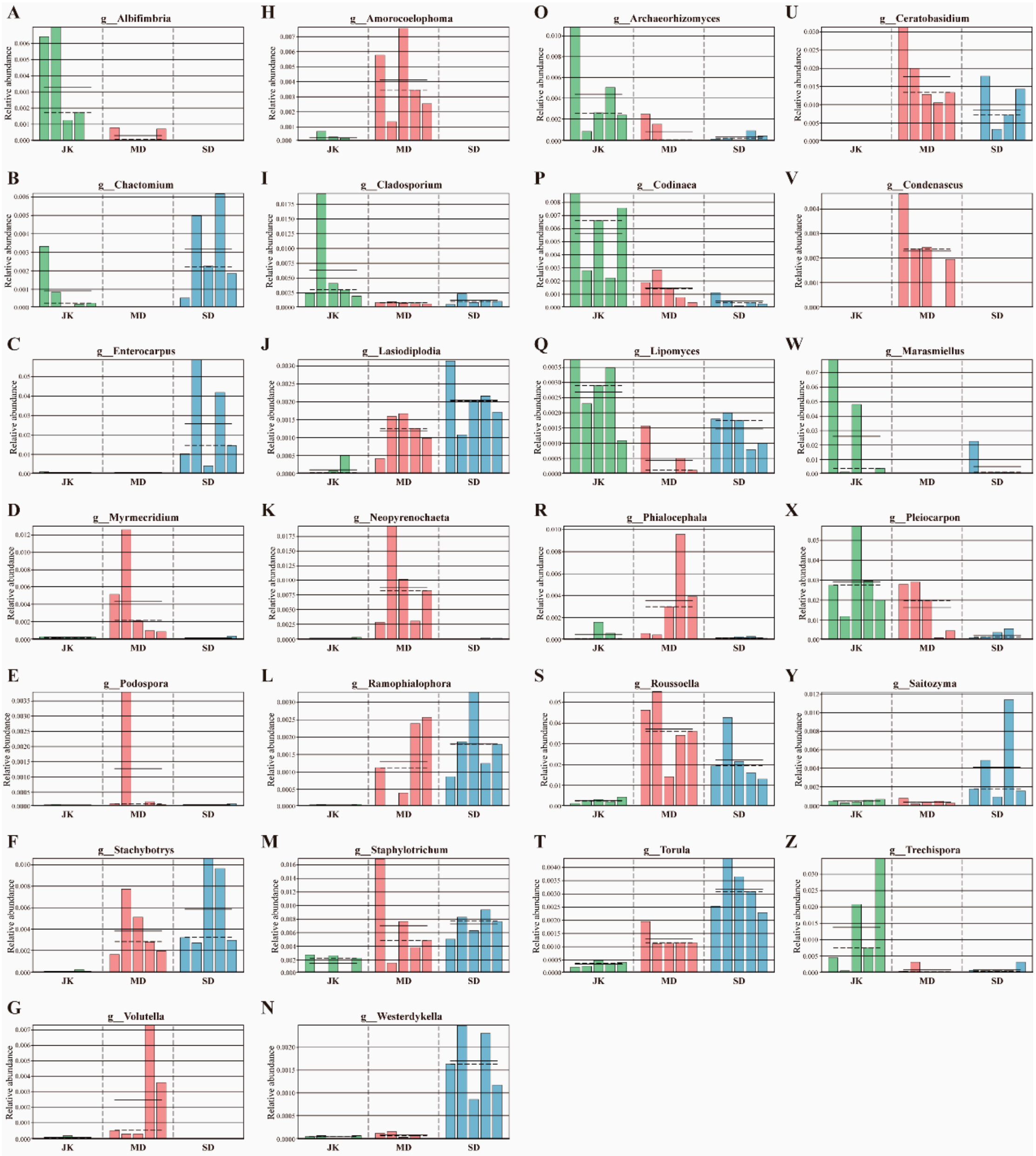

Figure S7. Statistical comparison results of the relative abundance of signature fungi at the genus level.

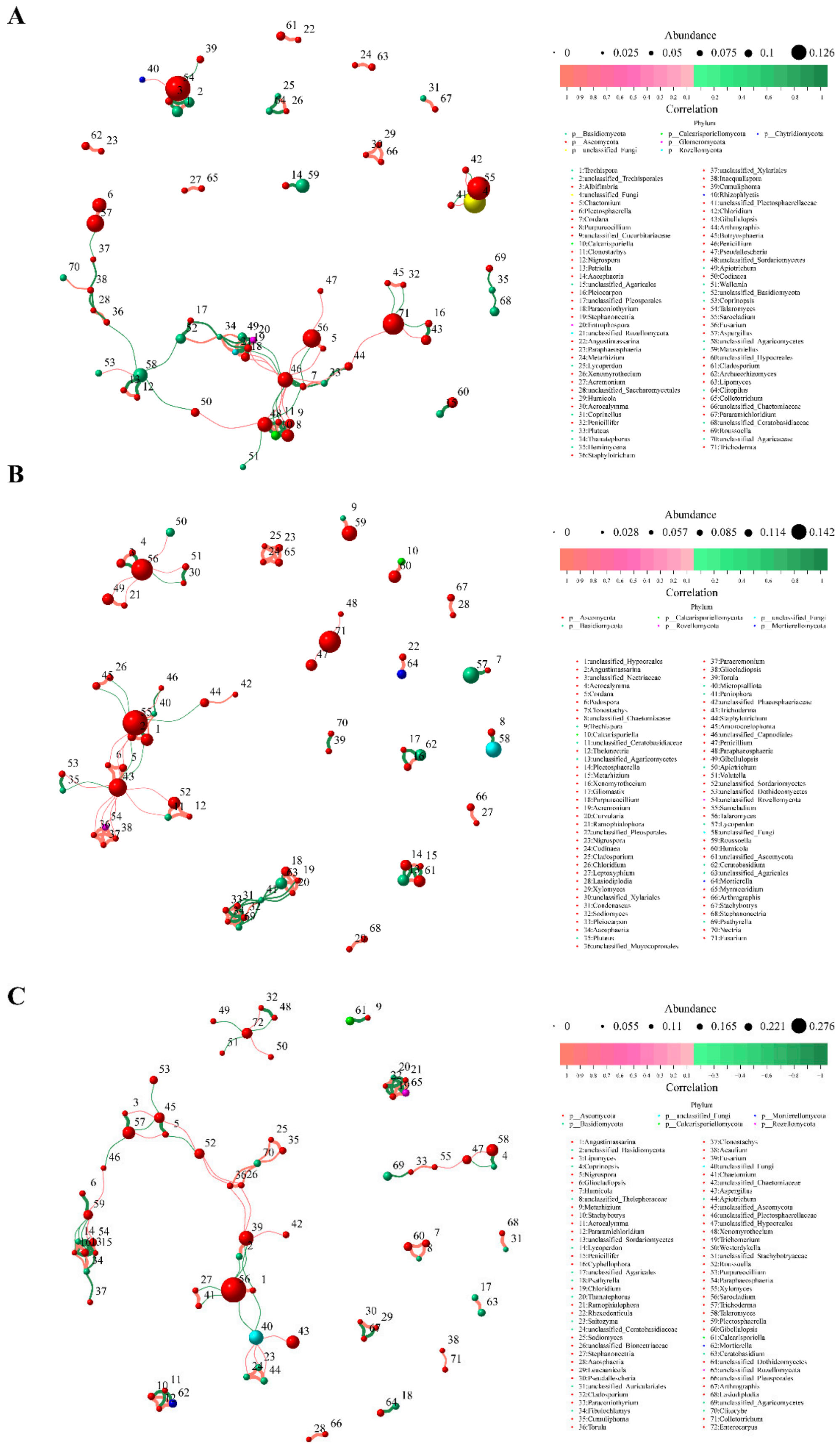

Figure S8. Correlation network of fungal communities across different samples. (A) represents healthy areca palm (JK). (B) represents mildly diseased areca palm (MD). (C) represents severely diseased areca palm (SD).

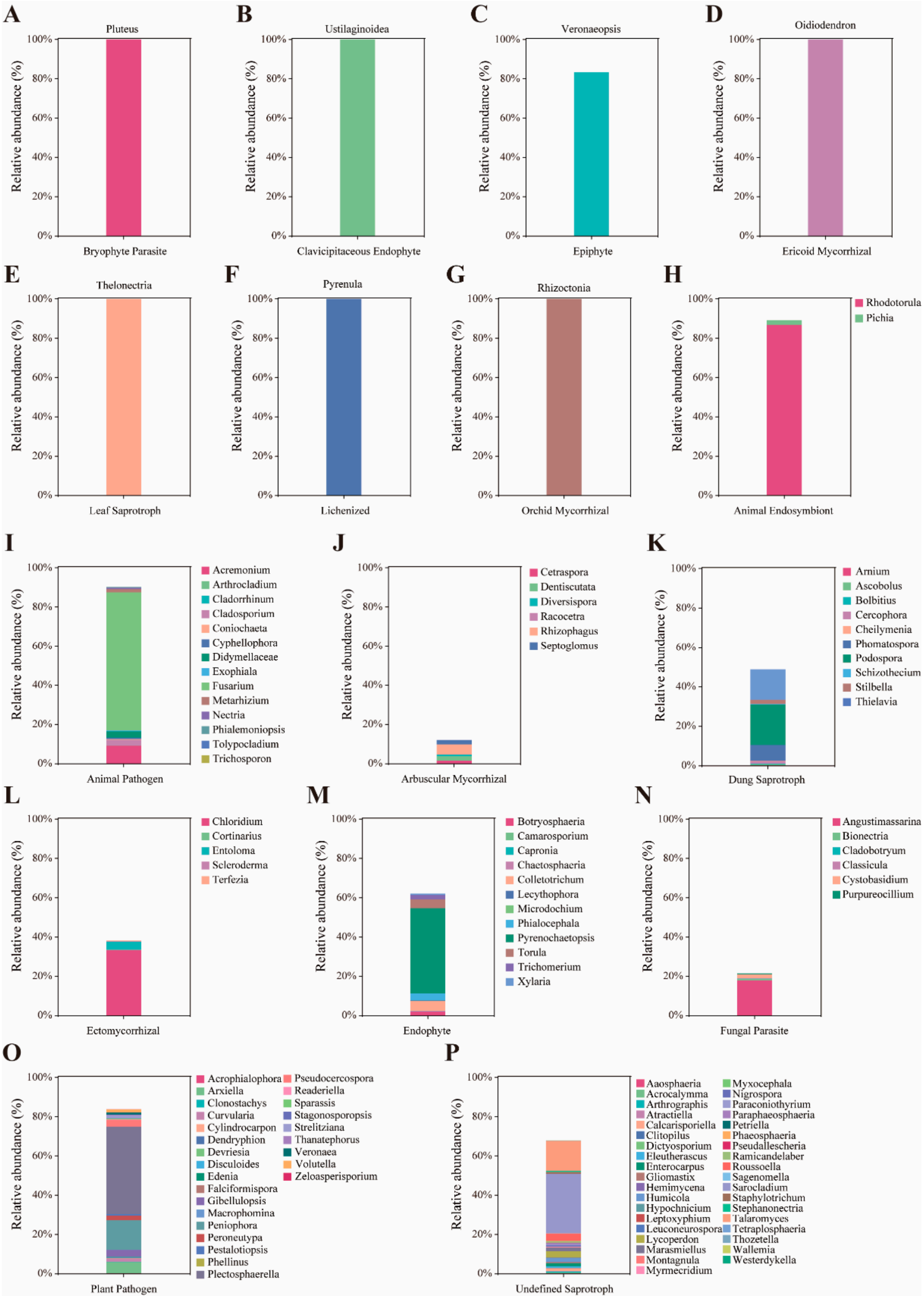

Figure S9. Genus-level biological function prediction. (A)-(P) The proportion of genera within each ecological functional group.
